# Bioinformatics analyses reveal the autophagy-related feature biomarkers in dilated cardiomyopathy with heart failure

**DOI:** 10.3389/fcvm.2025.1692768

**Published:** 2026-01-14

**Authors:** Jiayu Ren, Zhihan Li, Yue Wang, Ying Wang, Jing Li

**Affiliations:** 1 The Institute of Heart and Vascular Diseases, Second Affiliated Hospital of Dalian Medical University, Dalian, Liaoning, China; 2 Department of Physiology, College of Basic Medical Science, Dalian Medical University, Dalian, Liaoning, China; 3 Department of Pathology, Second Affiliated Hospital of Dalian Medical University, Dalian, Liaoning, China

**Keywords:** dilated cardiomyopathy, heart failure, autophagy, bioinformatics, gene

## Abstract

**Objective:**

Dilated cardiomyopathy (DCM) is a major cause of heart failure (HF). In this study, we aimed to explore potential autophagy-related biomarkers associated with DCM with HF.

**Methods:**

The GSE17800 dataset was downloaded from GEO, and differentially expressed genes (DEGs) were identified. Autophagy-related DEGs (AR-DEGs) were obtained by merging DEGs with autophagy-related genes (ARGs) from HADb and HAMdb databases. Gene function enrichment analysis was performed using GO and KEGG. Hub genes were identified via protein-protein interaction (PPI) network analysis, with their expression and diagnostic values validated using the GSE21610 dataset. A doxorubicin (DOX)-induced cardiomyocyte injury model was established to evaluate hub gene expression *in vitro* and *in vivo* studies. Potential therapeutic small molecules targeting hub genes were screened via L1000FWD, and their binding affinity to targets was assessed by molecular docking.

**Results:**

In the GSE17800 dataset, a total of 45 AR-DEGs were identified by intersecting with ARGs from HADb and HAMdb. Through PPI network analysis, 7 hub genes were extracted: CDKN1A, CTSD, DDIT3, EP300, FN1, PKM, and SOD2. Further validation using the GSE21610 dataset showed that receiver operating characteristic (ROC) curve analysis confirmed CTSD and SOD2 had high diagnostic value for DCM with HF. Moreover, in both *in vitro* and *in vivo* DOX-induced cardiomyocyte injury models, DOX treatment resulted in upregulated CTSD expression and downregulated SOD2 expression. Additionally, small molecules targeting CTSD and SOD2 (e.g., QL-XII-47 and tipifarnib-P2) were identified as potential therapeutic candidates for DCM with HF.

**Conclusion:**

This study provides novel evidence that CTSD and SOD2 potently contribute to autophagy regulation in DCM with HF. These findings highlight their diagnostic potential for DCM with HF and lay a foundation for exploring targeted small-molecule therapies (e.g., QL-XII-47, tipifarnib-P2) to improve the disease's clinical management.

## 1 Introduction

Dilated cardiomyopathy (DCM) is a primary disorder of the myocardial muscle. It is characterized by left ventricular (LV) systolic dysfunction and LV enlargement, often accompanied by LV or biventricular systolic dysfunction (1). Common clinical features include a left ventricular ejection fraction (LVEF) typically below 50% and the presence of systolic dysfunction, which eventually progresses to heart failure (HF) (2). Notably, DCM is frequently associated with an increased risk of severe arrhythmia and even sudden cardiac death (3). Additionally, the incidence is higher in men than in women, with the age at onset from 20 to 60 years old. The most common etiology of DCM is idiopathic, which may be characterized by a genetic and familial predisposition and is related to various biological processes, including cell proliferation, apoptosis and autophagy (4).

Autophagy is a cellular adaptive response to pathological conditions such as hypoxia, starvation, and oxidative stress. As a conserved physiological and defensive process, it plays a pivotal role in cellular waste clearance, structural remodeling, and maintenance of cell survival (5). Recent studies have shown that autophagy is involved in the occurrence and development of various cardiovascular diseases. Allicin affects cardiomyocyte size and improves cardiac function by inhibiting excessive autophagy through the PI3K/Akt/mTOR signaling pathway (6). In pathological cardiac hypertrophy, miR-17-5p attenuates the expression of the mitochondrial fusion protein mitofusin 2 in cardiomyocytes by autophagy mechanisms, thereby influencing the process of cardiac hypertrophy (7). Throughout the lifespan, autophagy becomes an essential mediator in maintaining cardiac function and improving clinical prognosis, but its role varies depending on the different stages of the disease (8). Importantly, the autophagy-related biomarkers in DCM with HF remain unclear. Thus, identifying potential autophagy-associated biomarkers is critical for developing novel therapeutic strategies for the management of DCM with HF.

**Figure 1:**
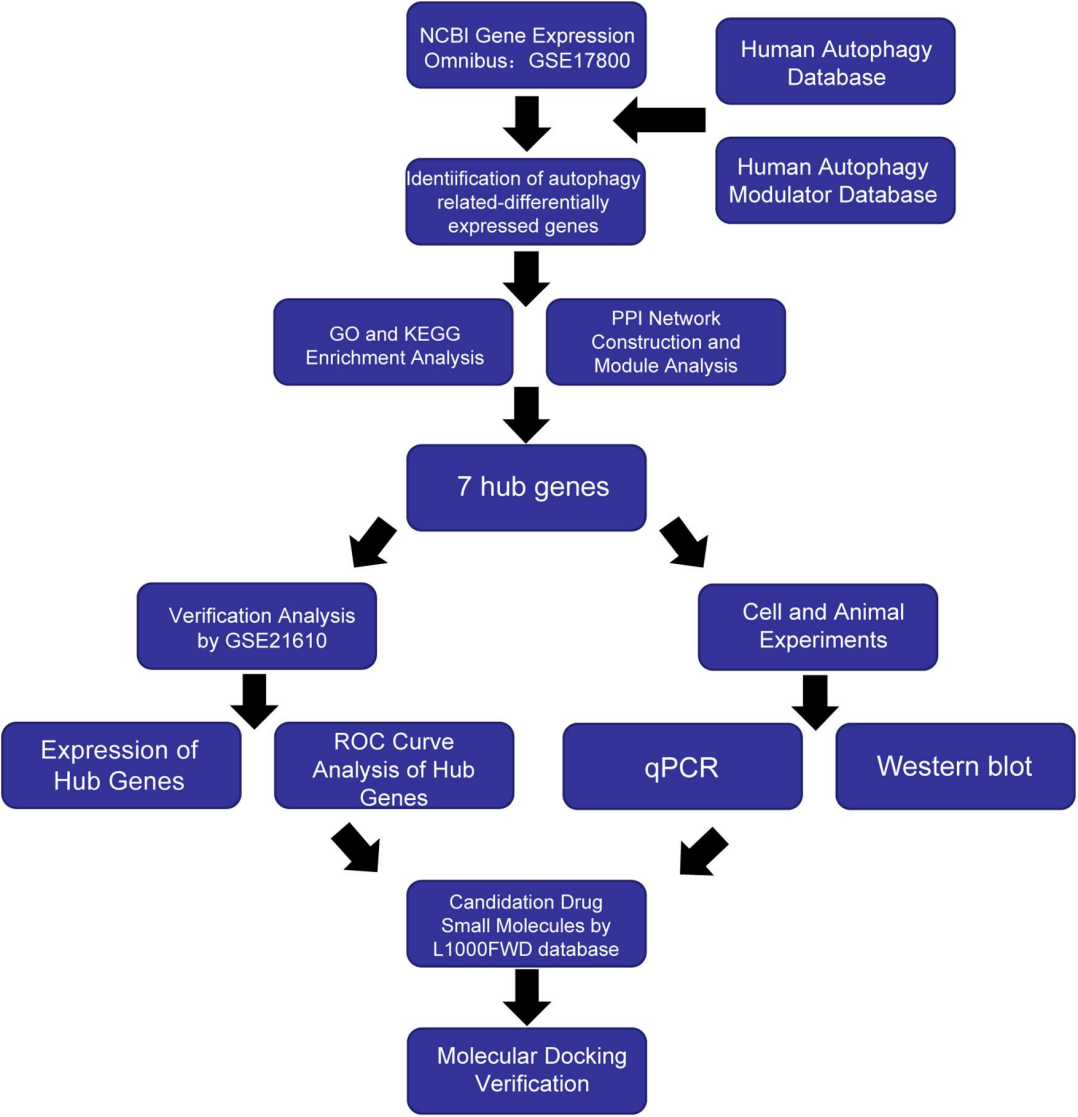
Workflow of the study.

The purpose of this study was to investigate the differential expression of autophagy-related biomarkers in DCM with HF by bioinformatics analysis, with the aim of providing novel evidence on the diagnosis and treatment methods. Figure 1 presents an overview of our analysis workflow.

## 2 Materials and methods

### 2.1 Dilated cardiomyopathy dataset acquisition

We downloaded the GSE17800 and GSE21610 datasets from the Gene Expression Omnibus (GEO) database (https://www.ncbi.nlm.nih.gov/geo/). The GSE17800 dataset (GPL570 platform) had 40 samples from DCM and 8 normal control samples. The GSE21610 dataset (GPL570 platform) included 21 DCM patients and 8 individuals with normal LVEF. Additionally, both the GSE17800 and GSE21610 datasets included DCM patients who met the following criteria: no prior medical intervention, LVEF < 45%, and HF symptoms classified as New York Heart Association (NYHA) Class II–IV. Detailed clinical information for patients in the GSE17800 dataset is provided in Supplementary Table S1.

### 2.2 Autophagy genes acquisition

Based on the Human Autophagy Database (HADb, http://www.autophagy.lu/index.html), 232 genes were identified. Similarly, 796 autophagy genes were obtained from the Human Autophagy Modulator Database (HAMdb, http://hamdb.scbdd.com) (9). A total of 803 autophagy-related genes (ARGs) were obtained as the autophagy gene set for this study after removing duplicate genes from the two.

### 2.3 Identification of differentially expressed genes (DEGs) in ARGs

The normalized expression matrix of microarray data was downloaded from the GSE17800 dataset. The “limma” package of the R software (version 4.0.1) was used to identify differentially expressed genes (DEGs). Genes exhibiting a *P* value < 0.05 and an absolute fold-change value ≥ 0.5 were designated as DEGs. Heatmap and volcano plot were performed using the “heatmap” and “ggplot2” packages of the R software. To obtain the set of autophagy-related differentially expressed genes (AR-DEGs), we intersected the 803 ARGs with the DEGs derived from the GSE17800 dataset. Venn plots were created by using the Sangerbox tool (http://www.sangerbox.com/) to visualize the overlap of genes (10).

### 2.4 Enrichment analysis of AR-DEGs

GO and KEGG pathway enrichment analysis was performed using the DAVID web service (11) (https://david.ncifcrf.gov/) by inputting the AR-DEGs. GO analysis consisted of cellular components (CC), biological processes (BP) and molecular functions (MF) (12).

### 2.5 Protein-protein interaction (PPI) network analysis of AR-DEGs

AR-DEGs employed the online database STRING (https://string-db.org/) to analyze the protein interaction network of common DEGs and correlation analysis conducted using Cytoscape software (13). The Molecular Complex Detection (MCODE) plug-in screened out key protein expression molecules (filter criteria: degree cut-off = 2; node score cut-off = 0.2; k-core = 2; max depth = 100). CytoHubba was used to identify important genes in this network as hub genes.

### 2.6 Validation of hub genes by analysing external dataset

We selected GSE21610 as the validation dataset to verify the 7 hub genes (14). Specifically, IBM SPSS Statistics 25 was used to generate receiver operating characteristic (ROC) curves and calculate the area under the ROC curve (AUC) (15). The AUC is a metric that combines the sensitivity and specificity to describe the intrinsic validity of a diagnostic test, an AUC > 0.7 is considered to have significant diagnostic significance, while a *p* < 0.05 indicated statistical significance. The R software packages ggplot2 and ggpubr were utilized to draw barplots for determining whether hub genes exhibited differential expression between the DCM with HF and the control groups.

### 2.7 Cell counting kit-8 assay

Human hybrid cardiomyocyte cells (AC16 cells, SSRCC Company, China) were seeded into a 96-well plate at the density of 5 × 10^3^ cell/well and exposed to varying concentrations of DOX (HY15142A, MCE, NJ, USA) for 24 h. Subsequently, 10 μL of CCK-8 reagent (HY-K0301, MCE, NJ, USA) was added to each well for 2 h of incubation. The absorbance of each well was measured at a wavelength of 450 nm using a microplate reader (SpectraMax 190, CA, USA).

### 2.8 Cell culture and treatment

AC16 cells were cultured in DMEM/F12 media (10% FBS, 1% penicillin/streptomycin) under a controlled atmosphere of 5% CO2 at 37 ℃. DOX was treated to AC16 cells to mimic cardiomyocytes injury caused by DCM *in vitro* study (16–19). AC16 cells were incubated with DOX at a dose of 5 μmol/L or with PBS for 24 h.

### 2.9 Quantitative PCR (qPCR)

The method of qPCR was described as previous (20). The sequences of the qPCR primers are shown in Supplementary Table S2.

### 2.10 Western blot

The method of western blot was described as previous (20). Antibodies against CTSD (21327-1-AP), SOD2 (66474-1-Ig), GAPDH (6004-1-Ig), Goat Anti-Rabbit IgG (SA00001-2) and Goat Anti-Mouse IgG (SA00001-1) were obtained from Proteintech (Chicago, USA). The protein intensities were analyzed using Image J software.

### 2.11 Animal experiments

Male C57BL/6J mice (8 weeks of age) were purchased from the Chinese Academy of Medical Sciences (Beijing, China). All mice were housed under standardized conditions: a 12 h light/dark cycle, controlled temperature (20–25 °C), and relative humidity (50 ± 5%). Throughout the experiment, the mice had free access to standard chow and sterile water. The mice were randomly divided into two groups and received intraperitoneal injections: one group was administered DOX at a total cumulative dose of 25 mg/kg, while the control group received an equivalent volume of normal saline. The injections were given five times over a 30-day period, with each injection delivering a single dose of 5 mg/kg via intraperitoneal route (21). All experimental procedures were approved by the Animal Care and Use Committee of Dalian Medical University and complied with the NIH Guide for the Care and Use of Laboratory Animals.

### 2.12 Candidate drugs identification and molecular docking

For drug discovery, we utilized the L1000FWD online database (https://maayanlab.cloud/l1000fwd/) (22) to identify candidate small-molecule drugs for DCM with HF. Specifically, we selected compounds that exhibited an opposite correlation to the hub genes. Based on the similarity score, *p*-value, comprehensive score and other factors, the relevant candidate drug molecules were selected. To evaluate the reliability of drug-target binding, molecular docking was performed between these candidate drugs and the target proteins CTSD and SOD2. The Structure Data File (SDF) structures of the candidate drugs were retrieved from PubChem database (https://pubchem.ncbi.nlm.nih.gov/), while the 3D structures of CTSD and SOD2 were downloaded from the RCSB Protein Data Bank (PDB) (https://www.rcsb.org/). Molecular docking between the candidate drugs and the target proteins (CTSD/SOD2) were performed using the CB-DOCK2 online tool (23). The docking site with the lowest Vina score was selected as the optimal binding mode.

### 2.13 Statistical analysis

Statistical analyses were performed using the ggpubr package of R software, with boxplots generated utilizing the ggplot2 package. Student's *t*-test was used to compare the differences between the two groups, while One-way ANOVA was utilized for the comparison of three or more groups. A *P* value of <0.05 was considered statistically significant.

## 3 Result

### 3.1 Identification of AR-DEGs

**Figure 2:**
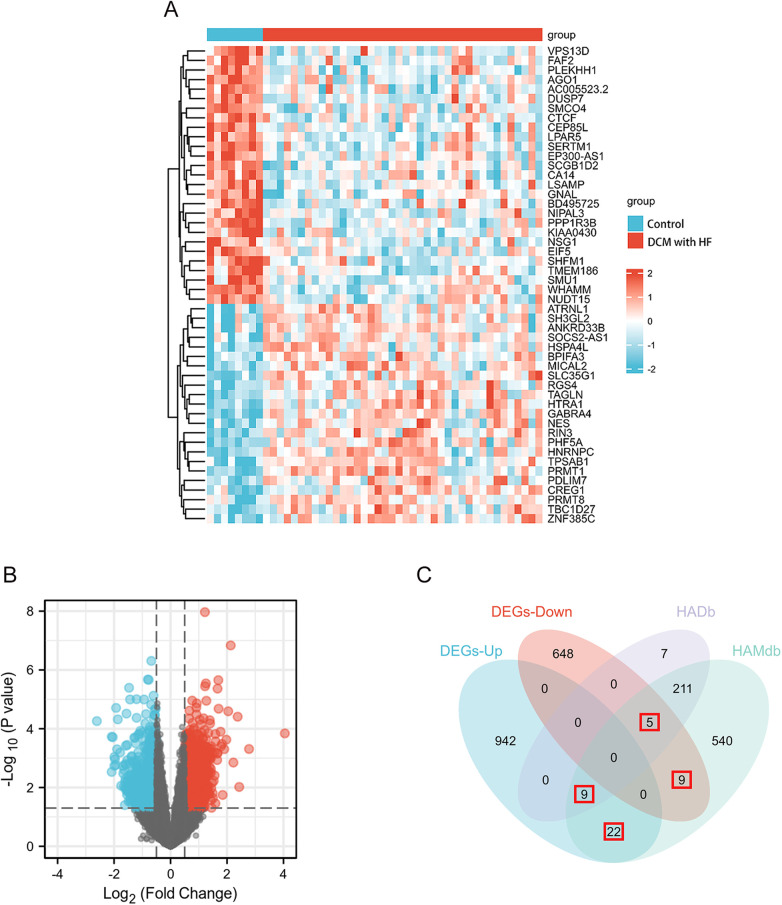
DEGs differential analysis of GSE17800 dataset and AR-DEGs were shown by venn diagram. **(A)** The heatmap shows the expression of the 50 most differentiated genes in the GSE17800 dataset. **(B)** Volcano plot of gene expression in the GSE17800 dataset. Red dots represent significantly up-regulated genes and blue dots represent significantly down-regulated genes. **(C)** Venn diagram shows 45 AR-DEGs in the GSE17800. The number of intersecting genes was marked in the red box. AR-DEGs, autophagy-related differentially expressed genes; DEGs-Up, differentially expressed up-regulated genes; DEGs-Down, differentially expressed down-regulated genes.

The GSE17800 dataset was used to screen for DEGs in DCM with HF. Based on the threshold of |log2FoldChange|≥ 0.5 and *p* value < 0.05, a total of 1635 DEGs were acquired, containing 973 upregulated genes and 662 downregulated genes. In addition, the heatmap (Figure 2A) shows the expression of the top 50 DEGs, and the volcano plot (Figure 2B) shows the distribution of DEGs. A total of 803 ARGs were obtained through two autophagy-related gene databases, HADb and HAMdb. As shown in the Venn diagram (Figure 2C), the intersection of these 803 ARGs with the DEGs from the GSE17800 dataset yielded 45 AR-DEGs.

### 3.2 Enrichment analysis

**Figure 3:**
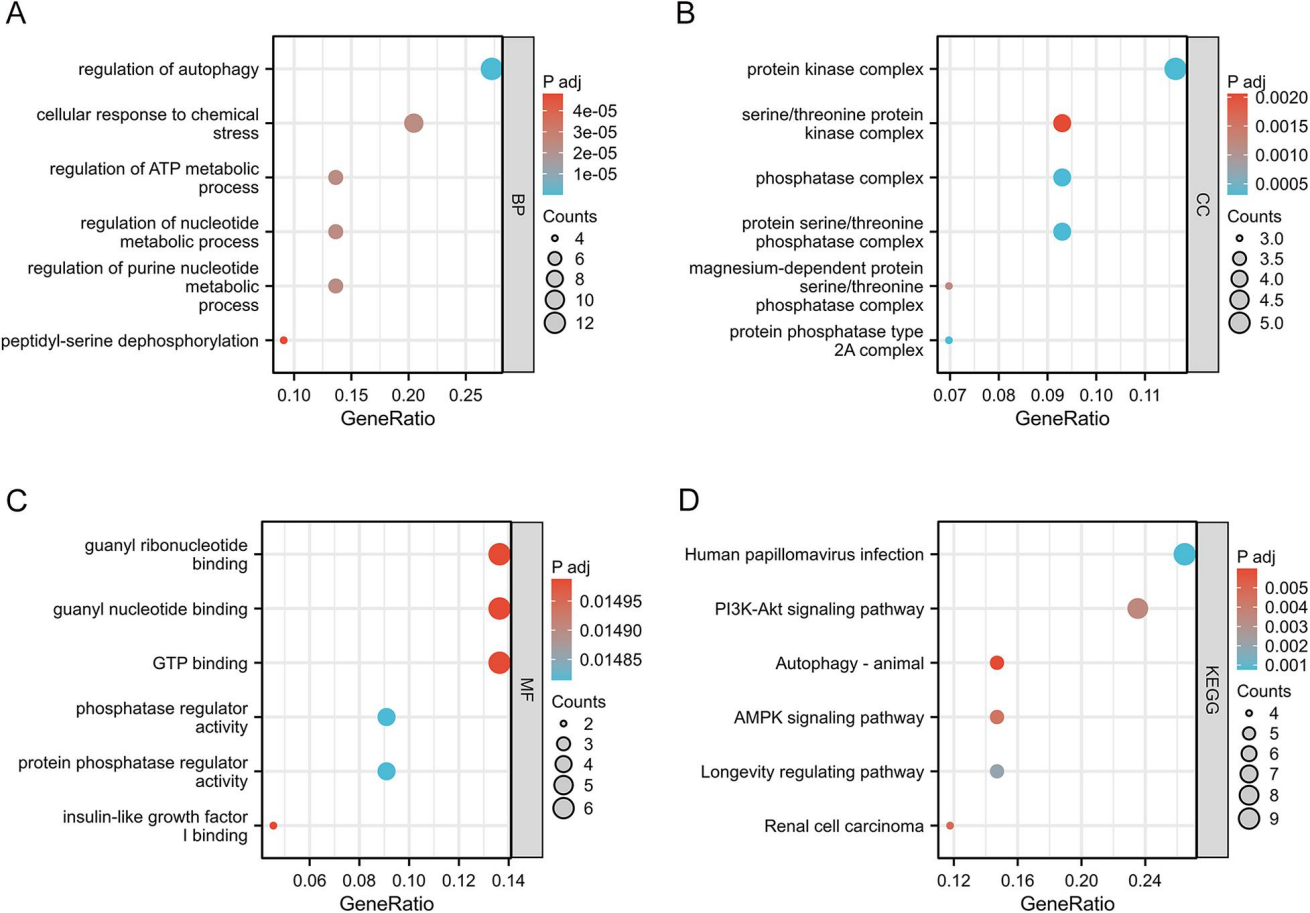
GO enrichment analysis and KEGG analysis of 45 AR-DEGs. **(A)** GO biological process (BP) enrichment results. **(B)** GO cell component (CC) enrichment results. **(C)** GO molecular function (MF) enrichment results. **(D)** KEGG pathway enrichment results. GO, gene ontology; KEGG, kyoto encyclopedia of genes and genomes; CC, cellular components; BP, biological processes; MF, molecular functions.

To evaluate the biological functions of these AR-DEGs, we used R software to perform GO and KEGG enrichment analyses. According to the results, the most significant GO-enriched terms involved regulation of autophagy, regulation of autophagy, cellular response to chemical stress, and regulation of ATP metabolic process (biological processes); protein kinase complex, serine/threonine protein kinase complex, and phosphatase complex (cellular components); and guanyl ribonucleotide binding, guanyl nucleotide binding, and GTP binding (molecular function) (Figures 3A–C). In the KEGG enrichment analysis, the AR-DEGs predominantly clustered around pathways including human papillomavirus infection, the PI3K-Akt signaling pathway, and autophagy-animal (Figure 3D). Overall, these enrichments point towards the involvement of AR-DEGs in autophagy and associated pathophysiological cascades.

### 3.3 PPI network analysis and hub genes identification

**Figure 4:**
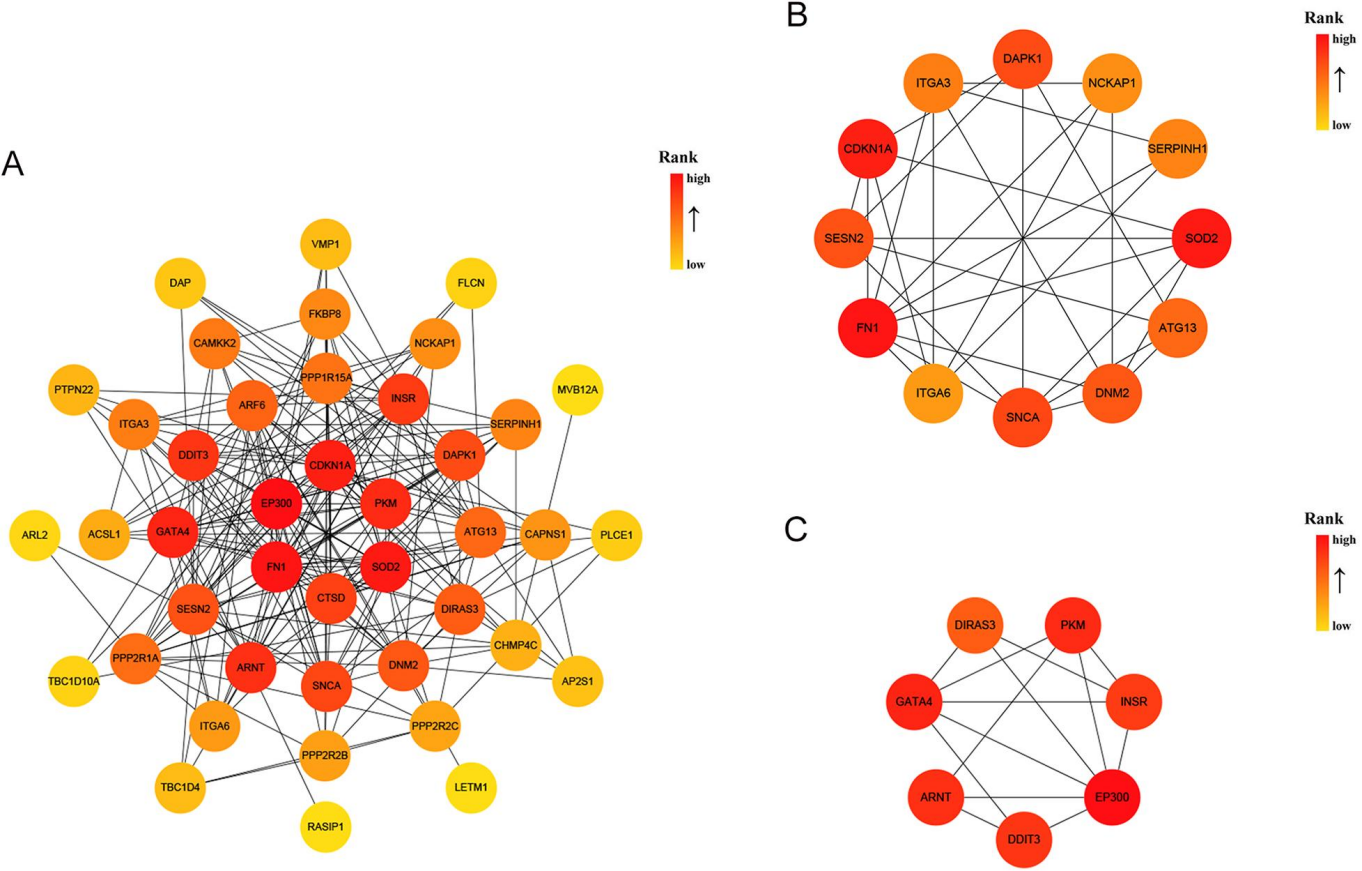
PPI analysis of 45 AR-DEGs. **(A)** The PPI among 45 AR-DEGs. Each node represented a protein, while each edge represented one protein–protein association. Two highly interconnected clusters **(B,C)** were identified in AR-DEGs by MCODE. From yellow to red, the darker the color, the higher the MCC score. PPI, protein–protein interaction; AR-DEGs, autophagy-related differentially expressed genes; MCODE, molecular complex detection; MCC, maximal clique centrality.

A DEG-encoded protein interaction network was constructed using STRING and visualized using Cytoscape, and it was composed of 42 nodes and 201 edges (Figure 4A). The MCODE plugin was used to identify gene cluster modules from the PPI network (Figures 4B,C). Cluster 1 exhibited the highest score (score: 5.455, 12 nodes and 30 edges), followed by Cluster 2 (score: 5, 7 nodes and 15 edges). Subsequently, the CytoHubba plugin was employed to identify hub genes. By intersecting the results of the four algorithms of CytoHubba, that is, MCC, MNC, EPC and Degree, 7 hub genes were identified, namely, CDKN1A, CTSD, DDIT3, EP300, FN1, PKM, and SOD2. These genes were the most essential biomarkers in the PPI network and are postulated to play a pivotal role in the pathogenesis of DCM with HF.

### 3.4 Verification of hub genes by GSE21610

**Figure 5:**
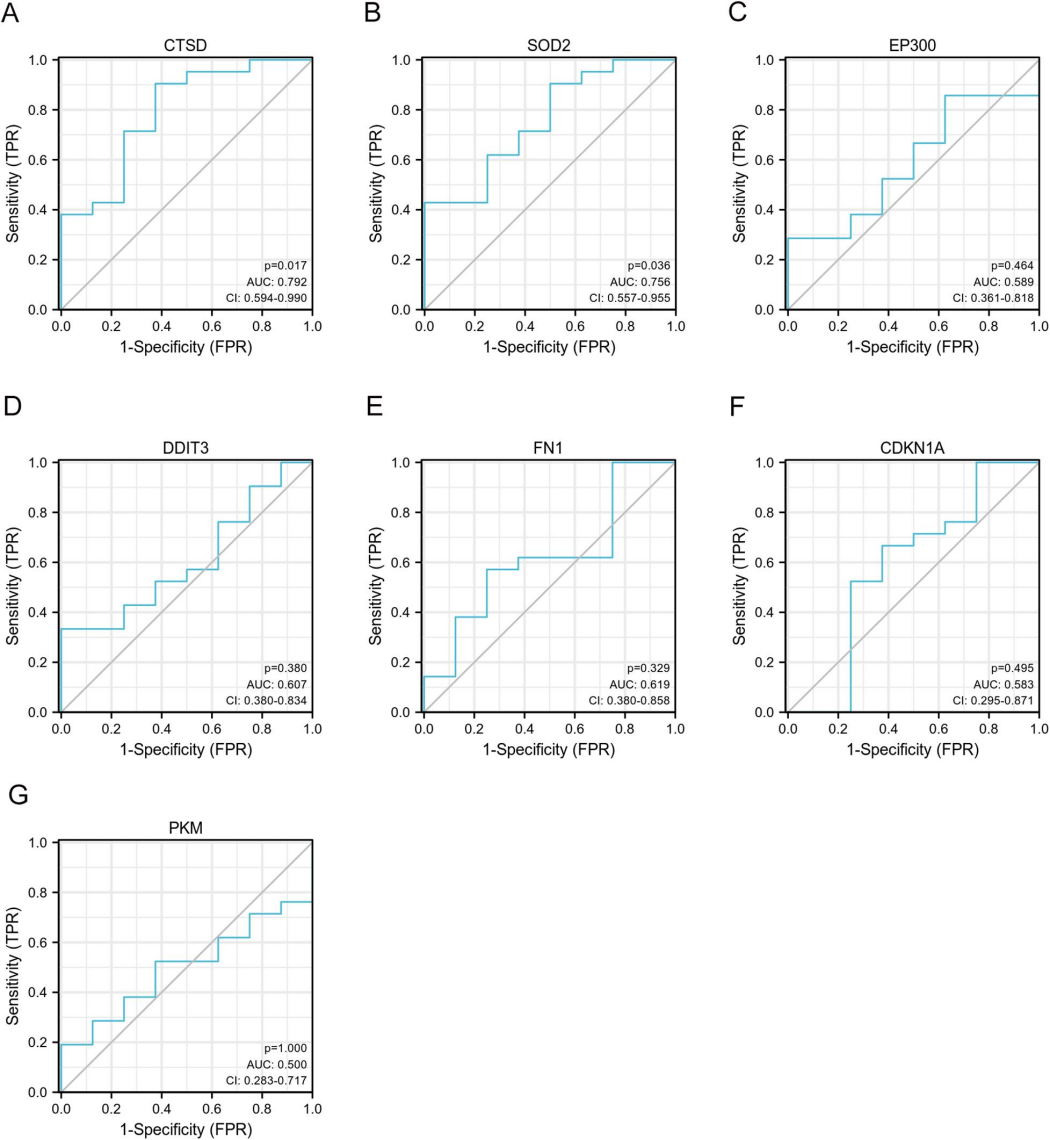
Validation of ROC curves of 7 specifically expressed hub genes by GSE21610. **(A–G)** The ROC curves of CTSD, SOD2, EP300, DDIT3, FN1, CDKN1A and PKM.

**Figure 6:**
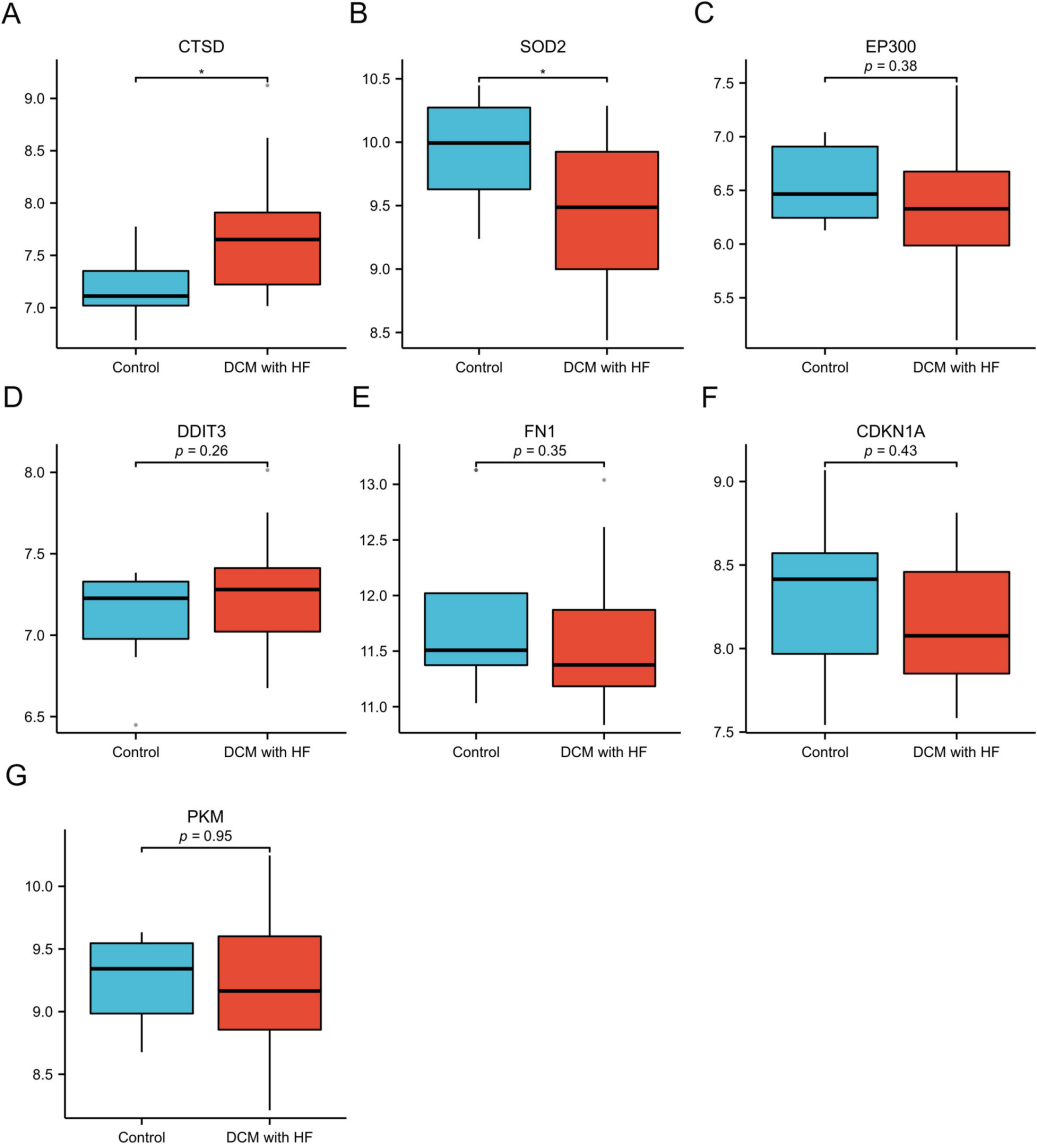
Validation of expression of 7 specifically expressed hub genes by GSE21610. **(A–G)** The expression of CTSD, SOD2, EP300, DDIT3, FN1, CDKN1A and PKM. *p < 0.05 vs. control group.

We selected GSE21610 as the validation dataset to verify the expression levels of the 7 hub genes. The GSE21610 dataset (GPL570 platform) contains 21 DCM with HF samples and 8 control samples. This dataset has been cited in numerous researches and is widely recognized. ROC curve analysis of the GSE21610 validation set yielded the AUC values for each hub gene (Figures 5A–G): (AUC = 0.792, *p* < 0.05), SOD2 (AUC = 0.756, *p* < 0.05), EP300 (AUC = 0.589), DDIT3 (AUC = 0.607), FN1 (AUC = 0.619), CDKN1A (AUC = 0.583) and PKM (AUC = 0.5). Notably, AUC > 0.7 is a commonly recognized criterion for assessing significant diagnostic value in clinical research. Based on this, only CTSD and SOD2 met this threshold, confirming their robust diagnostic potential for DCM with HF. This compelling data underscores CTSD and SOD2 as pivotal biomarkers, possessing enhanced diagnostic significance in DCM with HF. The detailed specifity and sensitivity of hub genes in Supplementary Table S3. Furthermore, hub gene expression levels offered additional supporting evidence (Figures 6A–G): compared with control samples, CTSD was significantly upregulated and SOD2 was significantly downregulated in DCM with HF samples (both *P* < 0.05). In contrast, the remaining hub genes (EP300, DDIT3, FN1, CDKN1A, and PKM) showed no statistically significant differences in expression between the two groups.

### 3.5 Verification of AR-DEGs *in vitro* and *in vivo*

**Figure 7:**
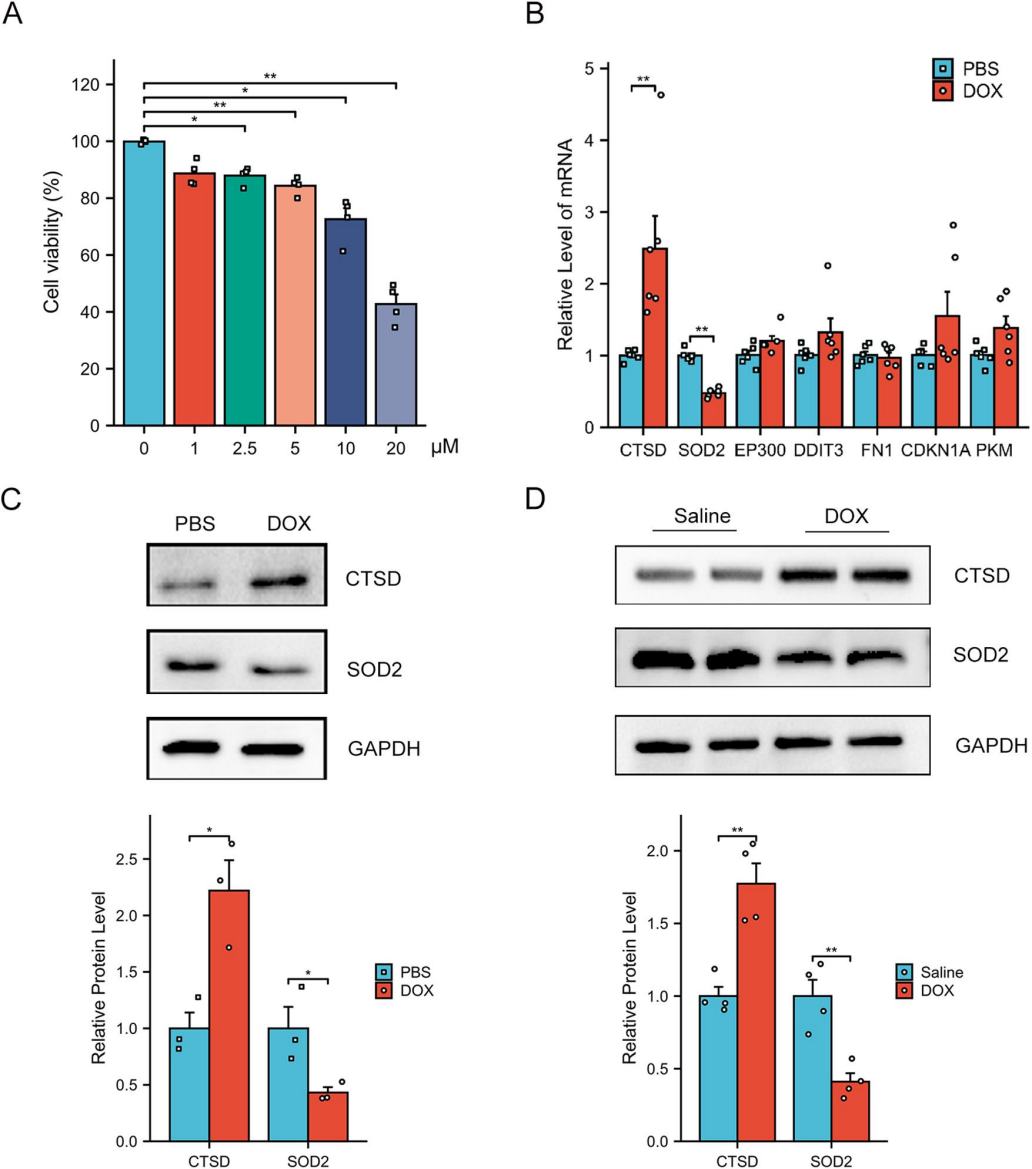
Verification of the AR-DEGs *in vitro* and *in vivo*. **(A)** Dose-dependent Dox-induced cardiac cell death was ascertained by CCK8 assay (*n* = 4). **(B)** qPCR analysis of CTSD, SOD2, EP300, DDIT3, FN1, CDKN1A and PKM in AC16 cells with PBS or DOX treatment (*n* = 6). The data are normalized to the GAPDH content. **(C)** Western blot analysis of CTSD and SOD2 in AC16 cells and quantification of the relative protein levels (*n* = 3). **(D)** Western blot analysis of CTSD and SOD2 in heart tissues and quantification of the relative protein levels (*n* = 4) *p < 0.05, **p < 0.01 versus control group.

To confirm the expression of hub genes *in vitro* experiments, we first established an *in vitro* model of cardiomyocyte injury mimicking DCM by treating AC16 cells with DOX (24). A CCK-8 assay was initially performed to evaluate AC16 cell viability following DOX treatment. As shown in Figure 7A, DOX significantly reduced the viability of AC16 cells in a dose-dependent manner. By integrating this result with findings from previous studies (25, 26), we selected 5 μM DOX as the subsequent concentration for subsequent *in vitro* experiments. Compared with the PBS-treated control group, DOX-treated AC16 cells exhibited significantly increased mRNA expression of CTSD and significantly decreased mRNA expression of SOD2 (Figure 7B). However, the mRNA levels of the other hub genes showed no statistically significant difference between the DOX- and PBS- treated groups. Furthermore, consistent with the mRNA results, the protein expression of CTSD was upregulated and that of SOD2 was downregulated in DOX-treated AC16 cells relative to the PBS control group (Figure 7C). Notably, we observed the same expression pattern of CTSD and SOD2 in our *in vivo* mouse experiments (Figure 7D).

### 3.6 Identification of the potential drugs

**Figure 8:**
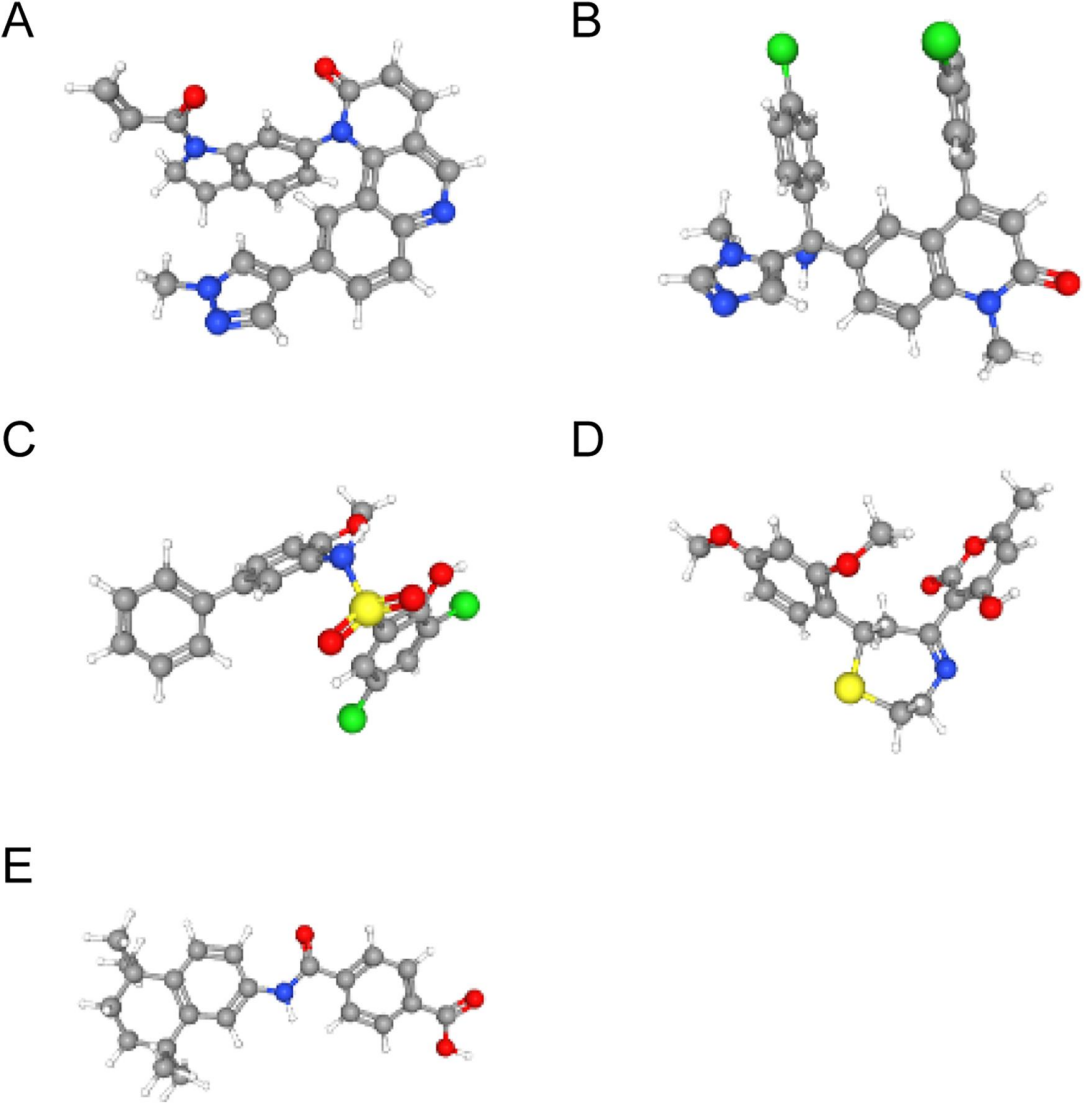
The structure of small-molecule compounds. **(A)** The 3D structure of QL-XII-47. **(B)** The 3D structure of tipifarnib-P2. **(C)** The 3D structure of CHEMBL-399379. **(D)** The 3D structure of KF-38789. **(E)** The 3D structure of tamibarotene.

**Figure 9:**
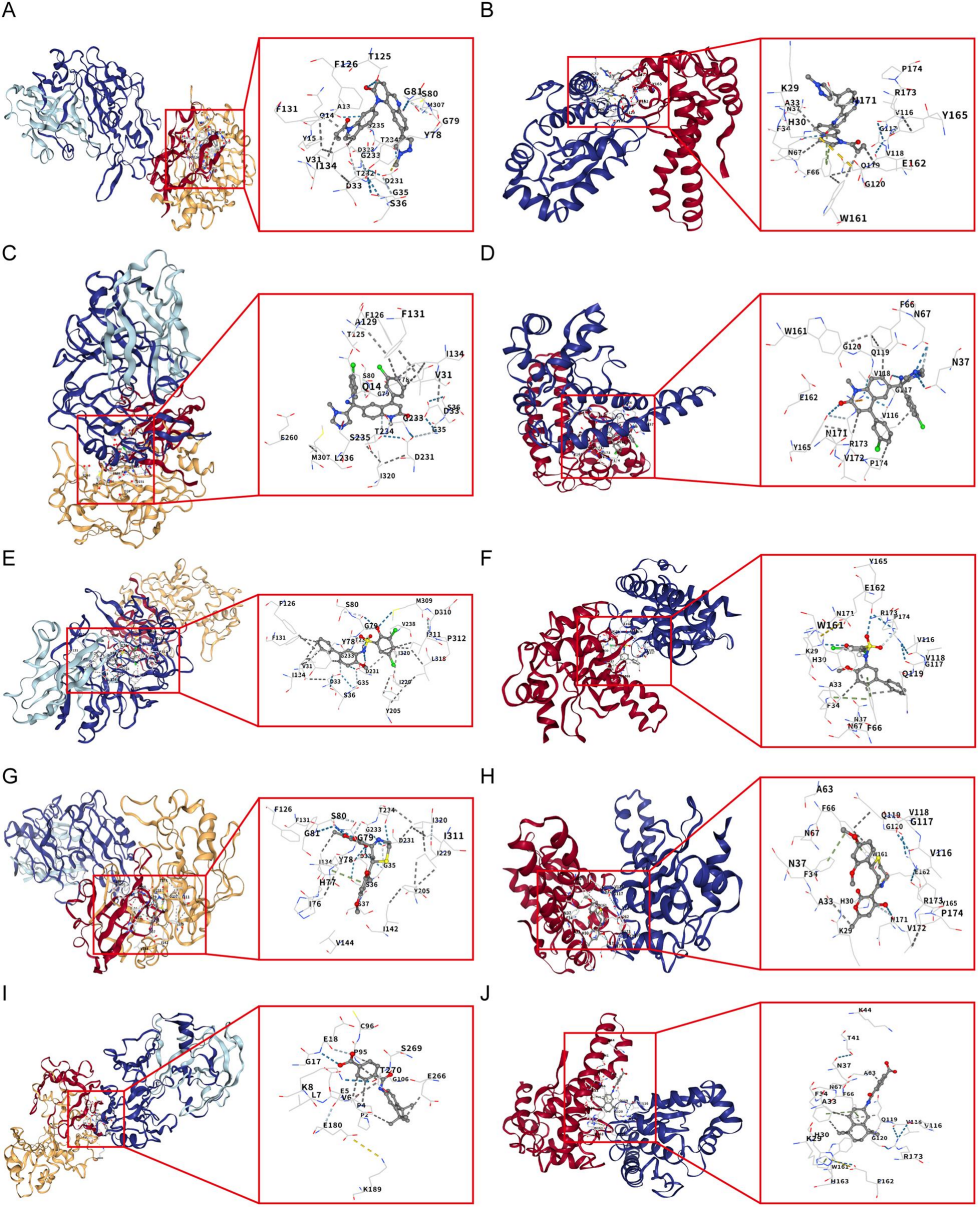
Molecular docking validation of candidate drugs with CTSD and SOD2. **(A)** Molecular docking between CTSD and QL-XII-47. **(B)** Molecular docking between SOD2 and QL-XII-47. **(C)** Molecular docking between CTSD and tipifarnib-P2. **(D)** Molecular docking between SOD2 and tipifarnib-P2. **(E)** Molecular docking between CTSD and CHEMBL-399379. **(F)** Molecular docking between SOD2 and CHEMBL-399379. **(G)** Molecular docking between CTSD and KF-38789. **(H)** Molecular docking between SOD2 and KF-38789. **(I)** Molecular docking between CTSD and tamibarotene. **(J)** Molecular docking between SOD2 and tamibarotene.

Using the L1000FWD database, we predicted potential regulatory drugs targeting CTSD and SOD2, ultimately identifying five small-molecule compounds as promising therapeutic candidates (Table 1). These compounds included: QL-XII-47, tipifarnib-P2, CHEMBL-399379, KF-38789 and tamibarotene, with their chemical structures were further described in Figure 8. Given the statistically favorable binding affinity scores of these compounds to CTSD and SOD2, we employed molecular docking to further evaluate their binding potential to the two target proteins. All molecular docking scores were less than −5 kcal/mol. These findings suggest that compounds such as QL-XII-47 and tipifarnib-P2, which exhibit particularly low binding energies with CTSD and SOD2, hold promising therapeutic potential for DCM with HF. The docking models of CTSD and SOD2 complexed with their top-scoring compounds are presented in Figure 9, with detailed docking parameters summarized in Table 1.

**Table 1:** Drug screening based on CTSD and SOD2.

Drug	Similarity score	*P* value	Z score	Combined score	CTSD (kCal·mol^−1^)	SOD2 (kCal·mol^−1^)
QL-XII-47	−1	0.000297	1.65	−5.83	−9.6	−9.1
Tipifarnib-P2	−1	0.000301	1.8	−6.35	−9.3	−8.7
CHEMBL-399379	−1	0.000344	1.76	−6.1	−8.6	−8.7
KF-38789	−1	0.000347	1.66	−5.75	−7.7	−7.7
Tamibarotene	−1	0.000355	1.59	−5.47	−8.3	−8.2

## 4 Discussion

DCM is a genetically susceptible myocardial disease characterized by systolic dysfunction. Among its clinical manifestations, DCM with HF poses a critical challenge in clinical management. Currently, heart transplantation remains the only reliable therapeutic option with potential curative effects for this condition (27). Autophagy plays an indispensable role in eukaryotic cells. Under physiological conditions, autophagy operates at a low basal level to maintain cellular homeostasis. In contrast, when cellular homeostasis is disrupted, autophagy is activated to support cell survival (28). Accumulating evidence indicates that aberrant autophagy is a pivotal factor in the pathogenesis of DCM (29). Furthermore, the pathological mechanism of autophagy in DCM with HF remains unknown, prompting increasingly interest in exploring distinctive biomarkers for this disease.

In mice with experimental DCM, cardiac dysfunction has been shown to be ameliorated by restraining autophagy through the mTOR-4EBP1 pathway (30). Previous research showed that the expression of Beclin 1, Atg5, and Atg7 was notably reduced in the hearts of DCM models (31). Specifically, activation of mTOR complex 1 inhibits autophagy and TFEB-dependent transcription of autophagy genes in DCM heart samples (32, 33). Currently, multiple researches have identified many autophagy biomarkers related to DCM through bioinformatics (10, 34, 35). However, little is known about autophagy-related genes in DCM with HF.

In our study, we identified 45 AR-DEGs by comparing biomarker expression profiles between the DCM with HF group and the control group. These 45 AR-DEGs genes were introduced into STRING for PPI analysis, and further subjected to MCODE module score analysis. Through these analyses, we obtained 7 autophagy-related hub genes that may be relevant to the progression of DCM with HF, namely: CTSD, SOD2, DDIT3, EP300, FN1, CDKN1A and PKM. Notably, numerous previous studies have confirmed that CDKN1A (36, 37), DDIT3 (38), FN1 (39) and EP300 (40) are closely related to DCM pathogenesis. However, the role of PKM in autophagy-related signaling pathways during DCM remains largely unknown. We further validated the expression patterns of these 7 hub genes using two approaches: the GSE21610 validation dataset, and *in vitro* and *in vivo* DCM models. Based on the cumulative evidence from our analyses, we hypothesized that the autophagy-related genes CTSD and SOD2 exhibit significant differential expression and possess diagnostic value in DCM with HF.

Cathepsin D (CTSD), a major member of the aspartic protease family in eukaryotic cells, plays a crucial part in various physiological processes, including cell proliferation, apoptosis, senescence, and autophagy. Kanamori et al. reported that cardiomyocytes from patients with DCM contained significantly more autophagic vacuoles (autophagosomes and autolysosomes) and higher CTSD expression levels, and notably, these patients experienced fewer cardiovascular events during the follow-up period (41). Consistent with these findings, our study also observed upregulated CTSD expression in DCM with HF. In addition, accumulating evidence has demonstrated that CTSD is closely associated with myocardial infarction (42), heart failure (43), and many kinds of cardiovascular diseases. Furthermore, ROC curve analysis of CTSD revealed that it exhibits a high diagnostic value in DCM with HF. Based on these collective observations, we considered CTSD as a novel and effective biomarker for the diagnosis of DCM with HF.

As a crucial member of the superoxide dismutase (SOD) family, manganese superoxide dismutase (MnSOD, SOD2) is primarily localized in mitochondria and plays a major role in regulating the intracellular redox balance. It has been verified that a homozygous missense variant of SOD2 can trigger severe DCM, heart failure and even death in human newborns. This pathogenic effect is mediated by increased levels of damaging reactive oxygen species (ROS) in the neonatal heart (44). Moreover, heart/muscle-specific SOD2-deficient (H/M-SOD2^−/−^) mice exhibit the typical pathological characteristics of DCM and further develop progressive HF. This phenotype arises from excessive ROS production in cardiomyocyte mitochondria, as mitochondrial redox homeostasis is disrupted without functional SOD2. Previous reports affirmed that H/M-SOD2^−/−^ mice serve as a reliable animal model of DCM combined with HF (45, 46). Consistent with these preclinical findings, a clinical study reported reduced SOD2 expression in patients with DCM (47). Collectively, these data underscore that SOD2 plays a vital role in susceptibility to DCM, with its dysfunction or reduced expression closely linked to DCM with HF pathogenesis.

Additionally, we screened the L1000FWD database for small molecules that exhibit an inverse correlation with CTSD and SOD2 expression, identifying five candidates: QL-XII-47, tipifarnib-P2, CHEMBL-399379, KF-38789 and tamibarotene. Results from molecular docking assays indicated that among these candidates, QL-XII-47 and tipifarnib-P2 formed the most stable binding complexes with CTSD and SOD2, respectively. Notably, previous studies have confirmed that tipifarnib reduces extracellular vesicles release and exerts a protective effect against heart failure progression (48). QL-XII-47, a Bruton's tyrosine kinase (BTK) inhibitor, has not been extensively explored for cardiovascular applications. However, preclinical evidence suggests that BTK inhibition can attenuate myocardial remodeling, fibrosis (49), and atherosclerosis (50, 51). Given these findings, the therapeutic potential of QL-XII-47 in cardiovascular diseases, particularly in DCM with HF, warrants further in-depth investigation. Collectively, these results suggest that the identified small molecules (especially QL-XII-47 and tipifarnib-P2) may represent promising therapeutic options for DCM with HF by directly targeting CTSD and SOD2 to modulate their expression or functional activity.

Our study has several limitations that should be acknowledged. Notably, we only verified the association between key biomarkers (e.g., CTSD and SOD2) and the progression of DCM with HF using C57BL/6J mouse models and AC16 cardiomyocytes, but we did not elucidate the specific molecular mechanisms through which CTSD and SOD2 regulate the pathogenesis of DCM with HF. Furthermore, the small-molecule drugs identified in our work are based on data analysis and virtual molecular docking results. Their functional effects have not been validated in either *in vivo* animal models or *in vitro* cell assays. Therefore, future studies should first clarify the specific mechanisms of CTSD and SOD2 in DCM with HF, and subsequently substantiate the therapeutic potential of these small molecules through well-designed animal experiments and cell-based assays.

## 5 Conclusion

Our work identified 45 potential ARGs associated with DCM with HF by bioinformatics analysis. In addition, we confirmed that CTSD and SOD2 serve as potential biomarkers, which may affect the occurrence and development of DCM with HF by regulating autophagic processes. These findings enhance our understanding of the molecular mechanisms underlying DCM with HF and provide a foundation for developing more effective diagnostic and therapeutic strategies for this disease.

## Data Availability

The original contributions presented in the study are included in the article/Supplementary Material, further inquiries can be directed to the corresponding author.
