# Supplementary material for: Bioinformatics analyses reveal the autophagy-related feature biomarkers in dilated cardiomyopathy with heart failure
Source: Front Cardiovasc Med. 2026 Jan 14;12:1692768. doi: 10.3389/fcvm.2025.1692768 (PMC12847351; doi:10.3389/fcvm.2025.1692768)
Supplement: Supplementary file 1 [file Datasheet1.docx]

**Supplementary Material**

**Bioinformatics Analyses Reveal the Autophagy-related Feature Biomarkers in Dilated Cardiomyopathy with Heart Failure**

Jiayu Ren^1,2†^, Zhihan Li^3†^, Yue Wang^1^, Ying Wang^1^ and Jing Li^1*^

1. The Institute of Heart and Vascular Diseases, Second Affiliated Hospital of Dalian Medical University, Dalian, Liaoning 116023, China.

2. The Department of Physiology, College of Basic Medical Science, Dalian Medical University, Dalian, Liaoning 116044, China.

3. The Department of Pathology, Second Affiliated Hospital of Dalian Medical University, Dalian, Liaoning 116023, China.

**Correspondence to: ***Jing Li, PhD, from the Institute of Heart and Vascular Diseases, Second Affiliated Hospital of Dalian Medical University, 467 Zhongshan Road, Dalian, Liaoning 116023, P.R. China. E-mail: [lijingfighting@163.com](mailto:lijingfighting@163.com)

Jiayu Ren^1,2†^ and Zhihan Li^3†^ These authors contributed equally to this work and share first authorship.

**Supplemental Tables**

**Table S1. Clinical information for the GSE17800 dataset.**

| **Sample** | **Gender** | **Age(y)** | **Group** | **EF %** | **LVEDD (mm)** |
| --- | --- | --- | --- | --- | --- |
| GSM444224 | male | 36 | DCM with HF | 39 | 72 |
| GSM444226 | male | 51 | DCM with HF | 38 | 69 |
| GSM444228 | male | 59 | DCM with HF | 29 | 84 |
| GSM444230 | male | 52 | DCM with HF | 35 | 72 |
| GSM444232 | male | 63 | DCM with HF | 35 | 75 |
| GSM444234 | male | 39 | DCM with HF | 39 | 64 |
| GSM444236 | male | 35 | DCM with HF | 38 | 73 |
| GSM444238 | male | 52 | DCM with HF | 42 | 73 |
| GSM444240 | female | 39 | DCM with HF | 30 | 52 |
| GSM444242 | female | 58 | DCM with HF | 34 | 69 |
| GSM444244 | female | 39 | DCM with HF | 25 | 64 |
| GSM444246 | female | 33 | DCM with HF | 43 | 61 |
| GSM444248 | female | 40 | DCM with HF | 28 | 72 |
| GSM444250 | male | 53 | DCM with HF | 30 | 72 |
| GSM444252 | male | 61 | DCM with HF | 39 | 62 |
| GSM444254 | female | 46 | DCM with HF | 30 | 75 |
| GSM444256 | male | 59 | DCM with HF | 32 | 61 |
| GSM444258 | male | 42 | DCM with HF | 35 | 74 |
| GSM444260 | female | 37 | DCM with HF | 40 | 66 |
| GSM444262 | male | 61 | DCM with HF | 34 | 69 |
| GSM444264 | male | 51 | DCM with HF | 41 | 78 |
| GSM444266 | male | 45 | DCM with HF | 41 | 62 |
| GSM444268 | female | 54 | DCM with HF | 26 | 87 |
| GSM444270 | female | 44 | DCM with HF | 42 | 63 |
| GSM444272 | male | 58 | DCM with HF | 40 | 69 |
| GSM444274 | male | 59 | DCM with HF | 20 | 80 |
| GSM444276 | male | 56 | DCM with HF | 30 | 78 |
| GSM444278 | male | 55 | DCM with HF | 27 | 58 |
| GSM444280 | male | 54 | DCM with HF | 29 | 72 |
| GSM444282 | male | 44 | DCM with HF | 35 | 62 |
| GSM444284 | male | 52 | DCM with HF | 38 | 77 |
| GSM444286 | male | 68 | DCM with HF | 32 | 71 |
| GSM444288 | female | 55 | DCM with HF | 22 | 63 |
| GSM444289 | male | 43 | DCM with HF | 25 | 73 |
| GSM444290 | male | 36 | DCM with HF | 22 | 87 |
| GSM444291 | male | 59 | DCM with HF | 38 | 63 |
| GSM444292 | male | 67 | DCM with HF | 41 | 67 |
| GSM444293 | female | 53 | DCM with HF | 28 | 56 |
| GSM444294 | female | 46 | DCM with HF | 35 | 69 |
| GSM444295 | male | 53 | DCM with HF | 24 | 78 |
| GSM444296 | male | 44 | Healthy control | 60 | 48 |
| GSM444297 | male | 20 | Healthy control | 57 | 47 |
| GSM444298 | male | 44 | Healthy control | 60 | 54 |
| GSM444299 | female | 59 | Healthy control | 50 | 49 |
| GSM444300 | female | 37 | Healthy control | 50 | 52 |
| GSM444301 | male | 28 | Healthy control | 62 | 56 |
| GSM444302 | male | 64 | Healthy control | 64 | 52 |
| GSM444303 | male | 49 | Healthy control | 75 | 53 |

EF: Ejection Fraction; LVEDD: Left ventricular end diastolic diameter.

**Table S2. Primers are used for quantitative real-time PCR analysis**

| **Gene** | **Forward Primer** | **Reverse Primer** |
| --- | --- | --- |
| h-CTSD | TGGAGAGGCAGGTCTTTGGGGA | TGTTGACGGAGATGCGGGGGTA |
| h-SOD2 | GTGGAGAACCCAAAGGGGAGTT | GTGGAATAAGGCCTGTTGTTCCTT |
| h-EP300 | CCTCGTATGCAACAGCCTTCC | CGGGCAGGAAGAACTAGACAT |
| h-DDIT3 | CCGGAATTCATGGAGCTTGTTCCAGCC | CGCGGATCCTCATGCTTGGTGCAGATTC |
| h-FN1 | ACAACACCGAGGTGACTGAGAC | GGACACAACGATGCTTCCTGAG |
| h-CDKN1A | CCGAAGTCAGTTCCTTGTGG | CATGGGTTCTGACGGACAT |
| h-PKM | GGAGAAACAGCCAAAGGGGACTA | CTGCCAGACTCCGTCAGAACTATC |
| h-GAPDH | GAAGGGCTCATGACCACAGT | GGATGCAGGGATGATGTTCT |

**Table S3. Sensitivity and specificity of hub genes**

| **Gene** | **Sensitivity** | **Specificity** |
| --- | --- | --- |
| CTSD | 0.90476 | 0.625 |
| SOD2 | 0.42857 | 1 |
| DDIT3 | 0.33333 | 1 |
| EP300 | 0.28571 | 1 |
| FN1 | 0.57143 | 0.75 |
| CDKN1A | 0.66667 | 0.625 |
| PKM | 0.19048 | 1 |
